# Next-generation sequencing analysis of a cluster of hepatitis C virus infections in a haematology and oncology center

**DOI:** 10.1371/journal.pone.0194816

**Published:** 2018-03-22

**Authors:** Kamila Caraballo Cortes, Magdalena Rosińska, Maciej Janiak, Małgorzata Stępień, Osvaldo Zagordi, Karol Perlejewski, Sylwia Osuch, Agnieszka Pawełczyk, Iwona Bukowska-Ośko, Rafał Płoski, Piotr Grabarczyk, Tomasz Laskus, Marek Radkowski

**Affiliations:** 1 Department of Immunopathology of Infectious and Parasitic Diseases, Medical University of Warsaw, Warsaw, Poland; 2 National Institute of Public Health–National Institute of Hygiene (NIPH–NIH), Warsaw, Poland; 3 Institute of Medical Virology, University of Zurich, Zurich, Switzerland; 4 Department of Medical Genetics, Medical University of Warsaw, Warsaw, Poland; 5 Department of Virology, Institute of Hematology and Transfusiology, Warsaw, Poland; Centers for Disease Control and Prevention, UNITED STATES

## Abstract

Molecular characterization of early hepatitis C virus (HCV) infection remains rare. Ten out of 78 patients of a hematology/oncology center were found to be HCV RNA positive two to four months after hospitalization. Only two of the ten patients were anti-HCV positive. HCV hypervariable region 1 (HVR1) was amplified in seven patients (including one anti-HCV positive) and analyzed by next generation sequencing (NGS). Genetic variants were reconstructed by Shorah and an empirically established 0.5% variant frequency cut-off was implemented. These sequences were compared by phylogenetic and diversity analyses. Ten unrelated blood donors with newly acquired HCV infection detected at the time of donation (HCV RNA positive and anti-HCV negative) served as controls. One to seven HVR1 variants were found in each patient. Sequences intermixed phylogenetically with no evidence of clustering in individual patients. These sequences were more similar to each other (similarity 95.4% to 100.0%) than to those of controls (similarity 64.8% to 82.6%). An identical predominant variant was present in four patients, whereas other closely related variants dominated in the remaining three patients. In five patients the HCV population was limited to a single variant or one predominant variant and minor variants of less than 10% frequency. In conclusion, NGS analysis of a cluster of HCV infections acquired in the hospital setting revealed the presence of low diversity, very closely related variants in all patients, suggesting an early-stage infection with the same virus. NGS combined with phylogenetic analysis and classical epidemiological analysis could help in tracking of HCV outbreaks.

## Introduction

The high level of intrahost and interhost hepatitis C virus (HCV) diversity results from the high error rate of RNA dependent RNA polymerase (RdRp) and fast replication of the virus. Consequently, HCV population represents a swarm of closely related variants called quasispecies. HCV variability enables the evasion of host adaptive immune responses and establishment of chronic infection, as well as drug resistance [1, 2]. Viral molecular diversity is often significantly reduced upon virus transmission to a new host (bottleneck effect) [3–6]. The bottleneck phenomenon may be affected by the size of the inoculum, HCV genotype, viral load and complexity of the virus population in the donor (number and frequency of variants), as well as recipient host factors, such as IL28B genotype [7, 8].

Studies on the early evolution of HCV following infection are rare due to the limited availability of clinical samples from the early stages of infection [9, 10]. In previous studies HCV intrahost diversity was analyzed using such well-established techniques such as DNA heteroduplex gel shift method [11] or bulk clonal sequencing [12]. However, their sensitivity with respect to minor variant detection is typically low [4, 13]. Novel methods suitable for in-depth analysis of quasispecies phenomenon were introduced such as single-genome analysis and next-generation sequencing (NGS) allowing for the evaluation of a wide spectrum of genetic variants, including those of minor frequency [5, 14, 15]. Despite some technical limitations, a reliable detection of variants constituting as little as 0.5% of the population became feasible [16].

In the present study we took advantage of a unique opportunity to investigate in-depth genetic diversity during early stages of infection, by analyzing a cluster of HCV infection among patients of a regional hematology and oncology center in Southern Poland. We investigated the diversity of hypervariable region 1 (HVR1) which represents a highly exposed fragment of envelope 2 glycoprotein playing a major role in HCV cell entry (receptor binding, membrane fusion) and is a major target for specific antiviral response (antibody shielding, epitopes for antibody responses) [17, 18]. Its variability facilitates immune evasion and reflects the immune pressure of the host [19].

Our study demonstrates the presence of very low HVR1 diversity in the early stage of viral infection. Since these variants were closely related, the patients were most likely infected from a common source.

## Materials and methods

### Patients

In November and December 2015 five clinically overt cases of acute hepatitis C infection were diagnosed among patients of a regional hematology and oncology center in Southern Poland. As all these patients had repeated hospital stays between August and October 2015, all patients hospitalized in this period in the same ward were contacted and asked to provide a blood sample for HCV infection screening and analysis. Out of 129 inpatients, 34 were already dead by the time of the study, 17 refused participation or could not be reached (including one patient from the initial cluster), and 78 provided both a sample and consent. Out of these tested individuals, HCV RNA was found in ten patients. Extensive epidemiological investigation did not identify the source of infection. Basic clinical and virological data on the study subjects are presented in Table 1.

**Table 1:** Clinical and virological characteristics of ten hospitalized patients infected with HCV 1b.

Patient ID	Sex	Age (years)	Anti-HCV^b^	Alanine aminotransferase activity (ALT) levels at the time of HCV-RNA detection [U L^-1^] (normal values 10–40 U L^-1^)	Viral load [IU mL^-1^]
1	F	26	negative	540	6.9x10^1^
2	M	49	negative	481	2.5x10^4^
3^a^	M	73	positive	N/A	1.6x10^7^
4^a^	F	80	negative	498	6.3x10^6^
5^a^	M	55	negative	751	1.6x10^7^
6^a^	M	66	negative	621	1.2x10^7^
7	F	68	negative	N/A	1.7x10^4^
8	F	63	negative	N/A	3.7x10^6^
9	M	64	positive	N/A	7.4x10^4^
10	M	54	negative	N/A	1.2x10^8^

N/A—not available

^a^ first patients to be diagnosed with clinically overt acute HCV infection

^b^Elecsys Anti-HCV assay (Roche Diagnostics, Mannheim, Germany)

Plasma samples from ten HCV RNA–positive, anti-HCV negative blood donors were used as controls for phylogenetic and sequence similarity comparisons. These controls were infected with the same HCV 1b subtype, and their infection was identified at the time of attempted donation.

The study was approved by the Bioethical Committee of the Medical University of Warsaw (Approval Number WUM AKBE/144/16) and Institute of Hematology and Transfusiology (Approval Number 55/2013) and all subjects and controls provided written informed consent.

### HVR1 amplification

HVR1 amplification was done as described in a previous publication [20]. In brief, total RNA was extracted from 250 μl of serum by a modified guanidinium thiocyanate-phenol/chlorophorm method using Trizol (Life Technologies, Carlsbad, CA, USA). Next, RNA was subjected to reverse transcription at 42°C for 60 minutes using AccuScript High Fidelity Reverse Transcriptase (Agilent Technologies, Santa Clara, CA, USA) and random hexamers. A region of 175 nt length encompassing HVR1 was amplified in two-step PCR using FastStart High Fidelity Taq DNA Polymerase (Roche, Indianapolis, IN, USA). Primers used for the first round amplification were as follows: 5′-CATTGCAGTTCAGGGCCGTGCTA-3′ (nt 1632–1610) and 5′-GGTGCTCACTGGGGAGTCCT-3′ (nt 1389–1408), according to the sequence of reference strain H77 (GenBank accession no. AF009606). Primers employed in the second PCR contained tags recognized by GS Junior sequencing platform, standard 10-nucleotide multiplex identifiers and target-complementary sequence [5′- TCCATGGTGGGGAACTGGGC-3′ (positions 1428–1447) and 5′-TGCCAACTGCCATTGGTGTT-3′ (positions 1603–1584)] [20].

### Pyrosequencing

Approximately 3×10^7^ DNA amplicons were subjected to emulsion PCR using the GS Junior Titanium emPCR Lib-A Kit (454 Life Sciences, Branford, CT, USA). Pyrosequencing was carried out according to the manufacturer’s protocol for sequencing amplicons using GS Junior (454 Life Sciences).

### Data analysis

Sequencing errors (mismatches, insertions and deletions) were corrected and haplotypes reconstructed using the program diri_sampler from the Shorah software suite (https://www1.ethz.ch/bsse/cbg/software/shorah) [21]. Haplotypes of posterior probability > 95% and represented by at least 10 reads were extracted with LStructure (https://github.com/ozagordi/LocalVariants/blob/master/src/LStructure.py). Based on pyrosequencing and reconstruction of a cloned HVR1 sequence [16] we were previously able to reliably detect variants constituting as little as 0.5% of the population and this cut-off was implemented in the current analysis. Subsequently, reconstructed haplotypes of frequency >0.5% were aligned to the consensus sequence (the most frequent sequence in all patients) and translated into amino acid sequences by MEGA (*Molecular Evolutionary Genetics Analysis)*, version 6.0 (http://www.megasoftware.net/) [22]. Phylogenetic trees were constructed according to the Maximum Likelihood method based on the Tamura-Nei model [23] using MEGA 6.0. We used the same approach in our previous studies [20, 24] and the superiority of Tamura-Nei model for the analysis of HVR1 was reported by others [25]. The robustness of tree topology was estimated by the bootstrapping method (resampling 1000 data sets) using MEGA 6.0. Genetic diversity parameters were assessed by DNA SP version 5 (http://www.ub.edu/dnasp/) and MEGA 6.0. Sequence similarity was compared using Clustal 2.1 Percent Identity Matrix (http://www.clustal.org/omega/) [26]. Furthermore, Highlighter from HIV.lanl.gov was used to visualize individual sequence polymorphisms [13].

## Results

In the present study the HVR1 amplification and molecular analysis were successful in seven out of ten HCV-infected patients. In three patients HVR1 could not be amplified mostly likely due to low viral load (patients 1 and 9), and mismatch between primers and particular viral strain (patient 10). An average of 4506 HVR1 sequence reads was obtained per sample (median 4149); (Table 2). Reads were reconstructed by SHORAH and, after implementation of the experimentally established 0.5% cut-off, one to seven HCV variants were retained per sample (mean 3.4, median 3.0). Mean nucleotide diversity was 0.032 (median 0.015) and number of nucleotide substitutions was 11.4 per patient (median 5.0). After translation to amino acid sequence, the number of variants ranged from one to three per patient (mean 2.4, median 3.0). The detailed data for each patient are presented in Table 2.

**Table 2:** Diversity parameters of HVR1 HCV variants in seven hospitalized patients infected with HCV 1b.

Patient ID	Number of NGS reads before filtering	Number of HVR1 nucleotide variants^a^	Number of HVR1 amino acid variants^a^	Number of nucleotide substitutions^a^^,^ ^b^	Nucleotide diversity (per site)^a^^,^ ^b^
2	3363	2	2	4	0.01515
3	3588	3	2	6	0.01705
4	4149	7	3	12	0.01705
5	4185	5	3	5	0.01061
6	5987	3	3	3	0.00852
7	6481	1	1	0	0.00000
8	3789	3	3	50	0.15333
MEAN (median)	4506 (4149)	3.4 (3.0)	2.4 (3.0)	11.4 (5.0)	0.03167 (0.01515)

^a^ >0.5% frequency cutoff

^b^ With respect to consensus sequence (the most frequent sequence in all patients)

**Fig 1:**
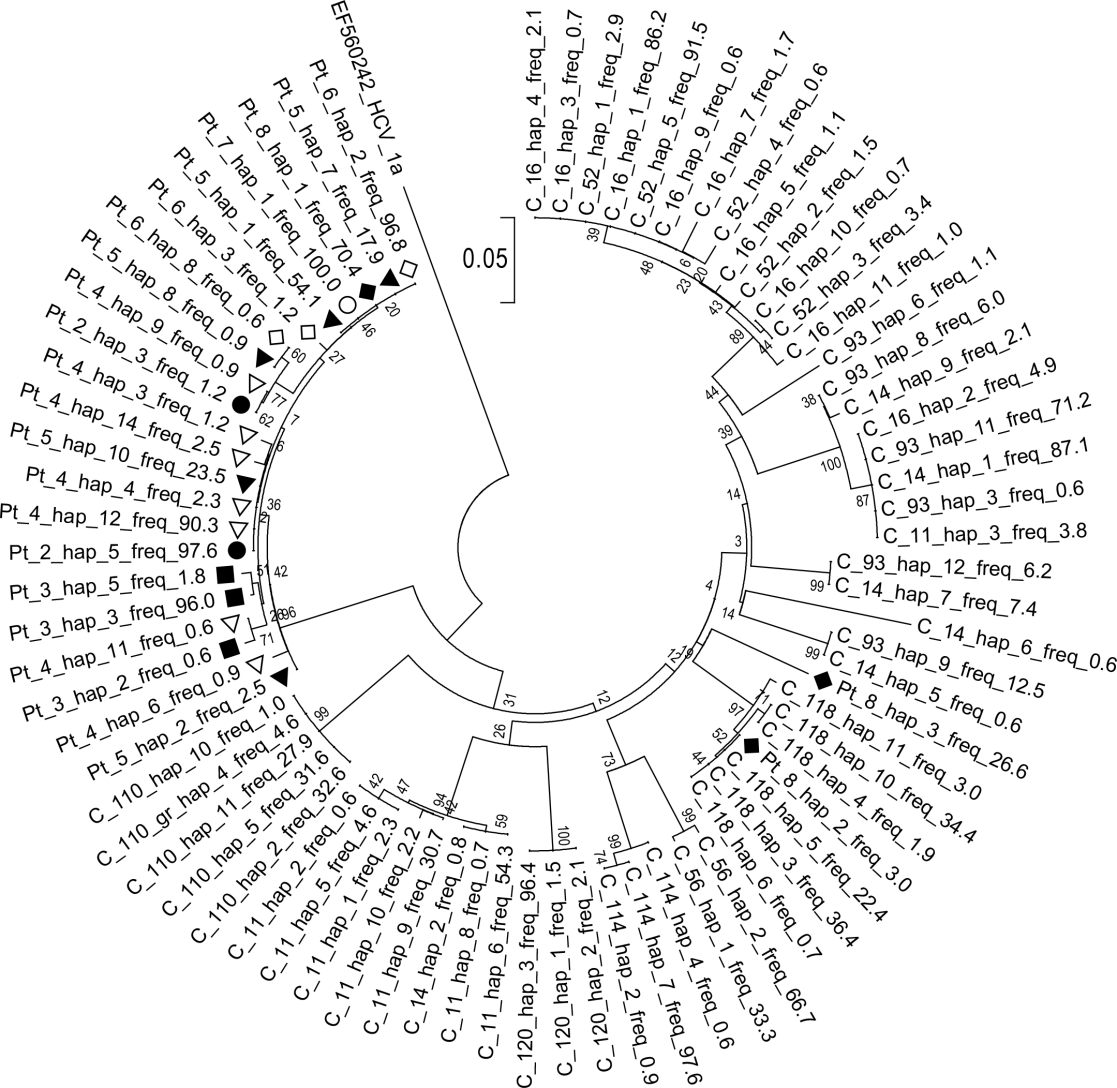
Phylogenetic analysis of HVR1 variants in seven hospitalized patients infected with HCV 1b and in ten unrelated controls. Variant frequencies are expressed as percent values and follow haplotype number. Pt denotes patients from the infection cluster. For clarity, each patient is marked with a different graphical symbol. Controls (C) came from the same geographic area and were infected with the same HCV subtype 1b. Bootstrap values obtained with 1000 replications are shown at the particular bifurcation points. HVR1 genotype 1a sequence (GenBank accession no. EF56024) has been used as an outgroup.

When all patients’ sequences were phylogenetically compared with sequences from ten unrelated controls, it was found that all the patients’ sequences clustered together except for two variants of lower frequency (26.6% and 3%) in patient 8, which clustered with variants from one control (C_118). Moreover, sequences derived from the cluster were interspersed with one another, with no evidence of clustering in individual patients (Fig 1).

**Fig 2:**
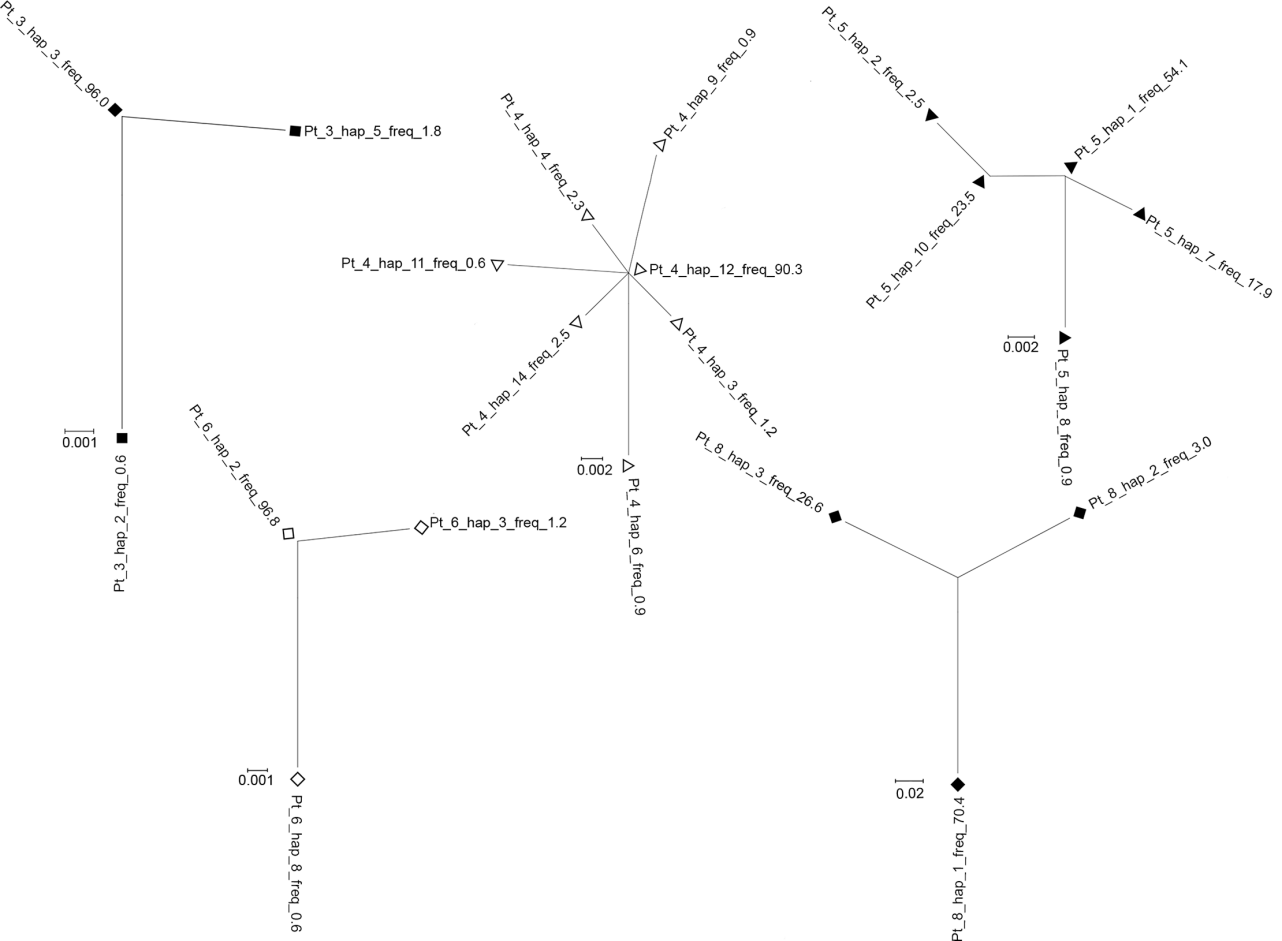
Phylogenetic analysis of HVR1 variants in hospitalized patients 3, 4, 5, 6 and 8. The trees from patients 3, 4 and 6 are consistent with infection with a single founder (star-like phylogeny). Variant frequencies are expressed as percent values and follow haplotype number. For clarity, each patient is marked with a different graphical symbol (corresponding to Fig 1).

Intrapatient phylogenetic trees could be constructed only for patients in whom at least three HVR1 variants were present (patients 3, 4, 5, 6 and 8; Fig 2). As seen, in patients 3, 4 and 6 the trees displayed star-like phylogeny while in patient 5 the tree was more complex, with higher number of clades. Nevertheless, the two dominant variants of 54.1% and 23.5% frequency differed by one substitution only. The variant of 54.1% frequency was identical to the consensus variant (the most prevalent variant in the analyzed patients) and the variant of 23.5% frequency harbored substitution which was also seen in variants from some other patients (patient 2, 3 and 4). In patient 8 all three variants show significant divergence from each other. In the remaining patients HVR1 population was comprised of only one (patient 7) or two variants (patient 2). In the latter the major variant prevailed at 97.6% of population.

**Fig 3:**
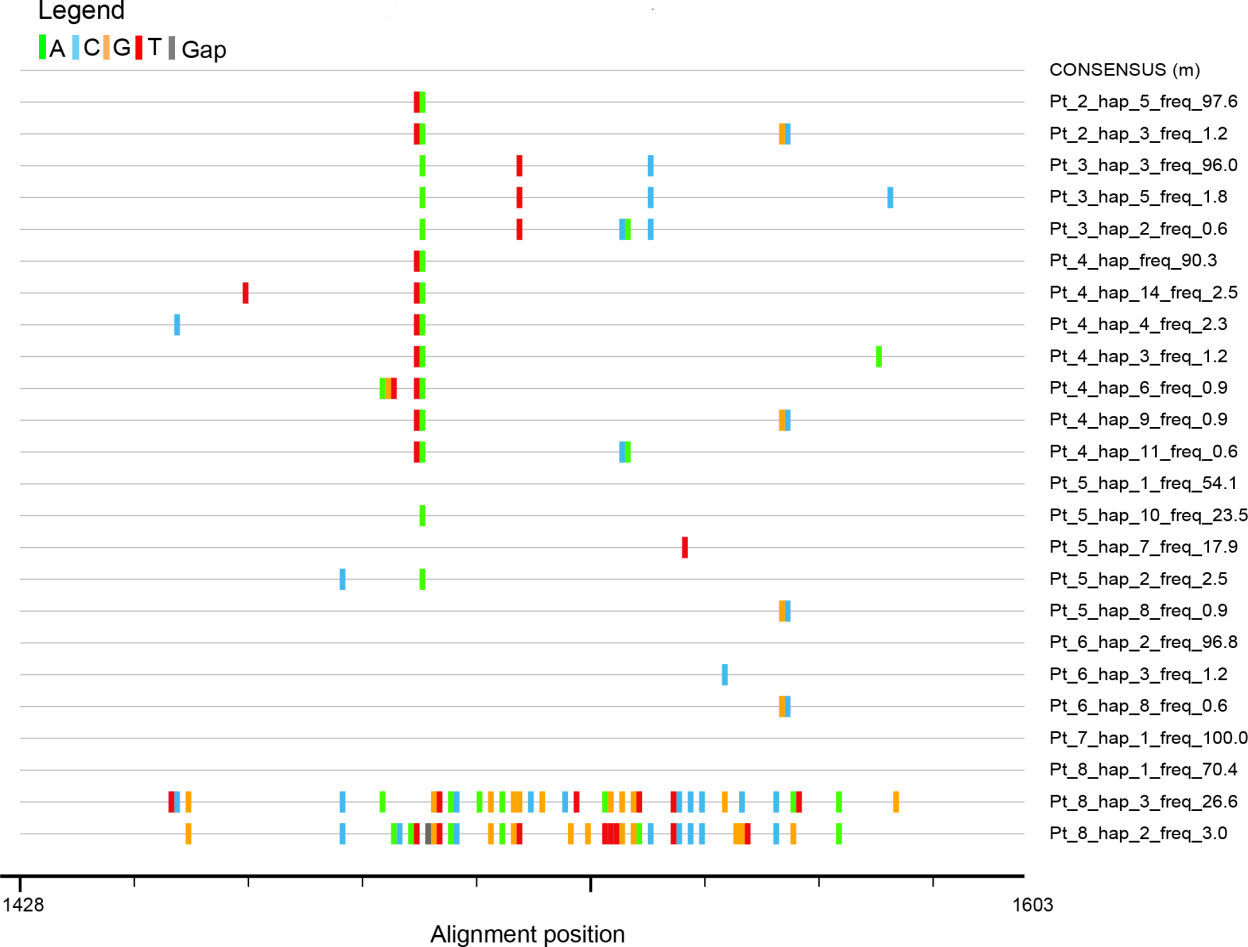
Highlighter plot showing differences (mismatches and gaps) of HVR1 HCV sequence variants in seven hospitalized patients. Variants are compared to consensus sequence for all sequences present in all patients. A, C, T and G mismatches and gaps are shown in green, blue, orange, red and gray, respectively. Nucleotide numbering follows the reference strain H77 (GenBank accession no. AF009606).

Sequence similarity analysis revealed that HVR1 sequences from the analyzed patients were more similar to each other (95.4% to 100.0%) than to the sequences derived from controls (64.8% to 82.6%). The only exception were two low frequency variants (26.6% and 3.0%) seen in patient 8. Similarity of the latter two variants to variants from the other six patients ranged from 79.0% to 82.8% while similarity to variants found in controls ranged from 67.6% to 98.3%. Comparison of sequences from all seven patients is shown on Fig 3.

Nucleotide sequence analysis revealed that the predominant variant was identical in patients 5, 6, 7 and 8 and a very similar variant was predominant in patients 2 and 4 (Fig 3). These two predominant variants differed only by two nucleotide substitutions (98.86% similarity). In patient 3 the predominant variants were slightly different from predominant variants in patients 2, 4, 5, 6, 7 and 8 (98.30% similarity).

**Fig 4:**
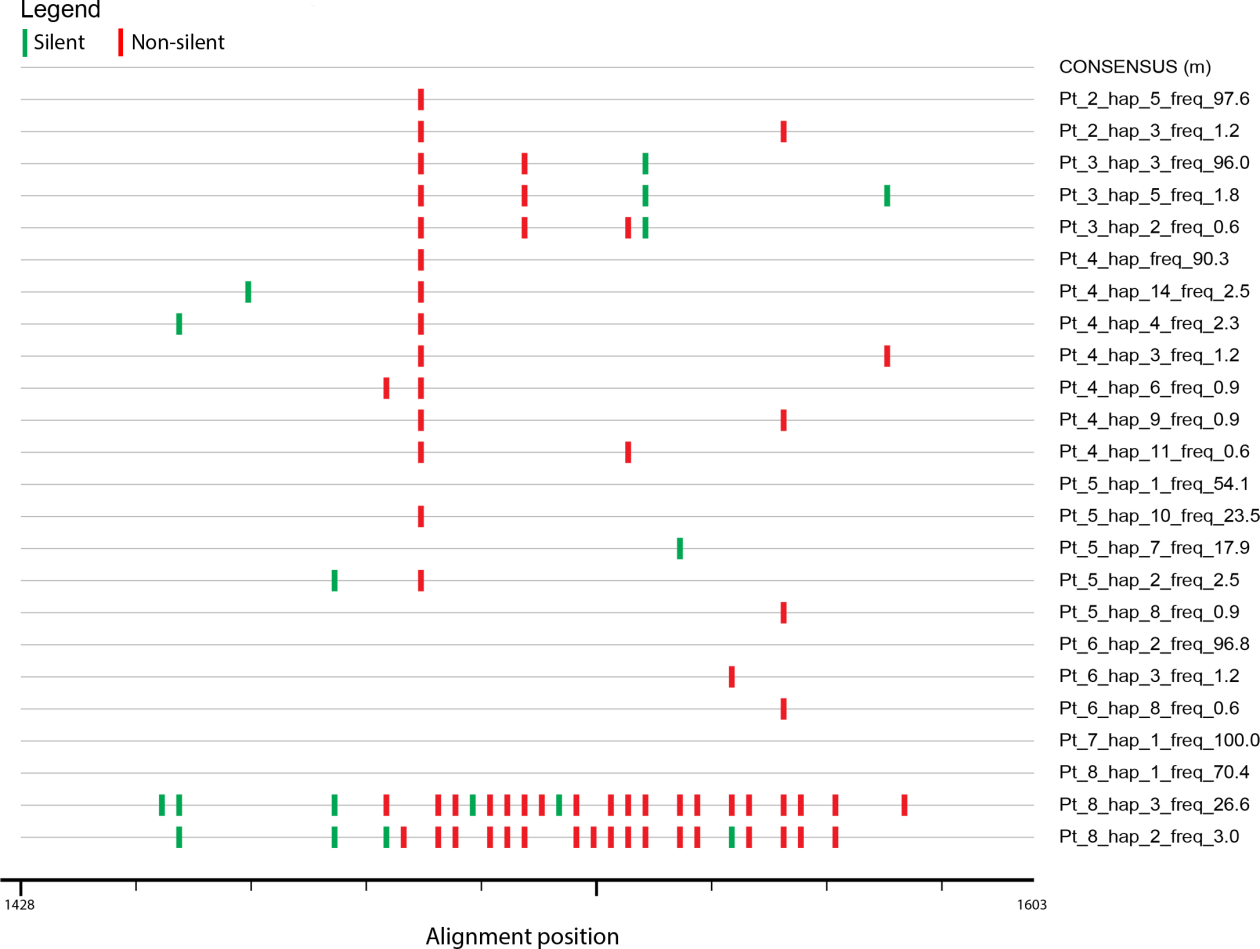
Highlighter plot showing silent and non-silent mutations of HVR1 HCV sequence variants in seven hospitalized patients from the infection cluster. Variants are compared to consensus sequence for all sequences present in all patients. Silent and non-silent mutations are shown in green and red, respectively. Nucleotide numbering follows the reference strain H77 (GenBank accession no. AF009606).

When the frequency structure of variants was analyzed it was found that in patients 2, 3, 4, 6 and 7 (71.4% of patients) the HVR1 populations were “narrow” (i.e. limited to single variant or to one predominant variant and minor variants of less than 10% frequency). When compared to consensus sequence, nucleotide substitutions were largely non-silent (Fig 4).

**Fig 5:**
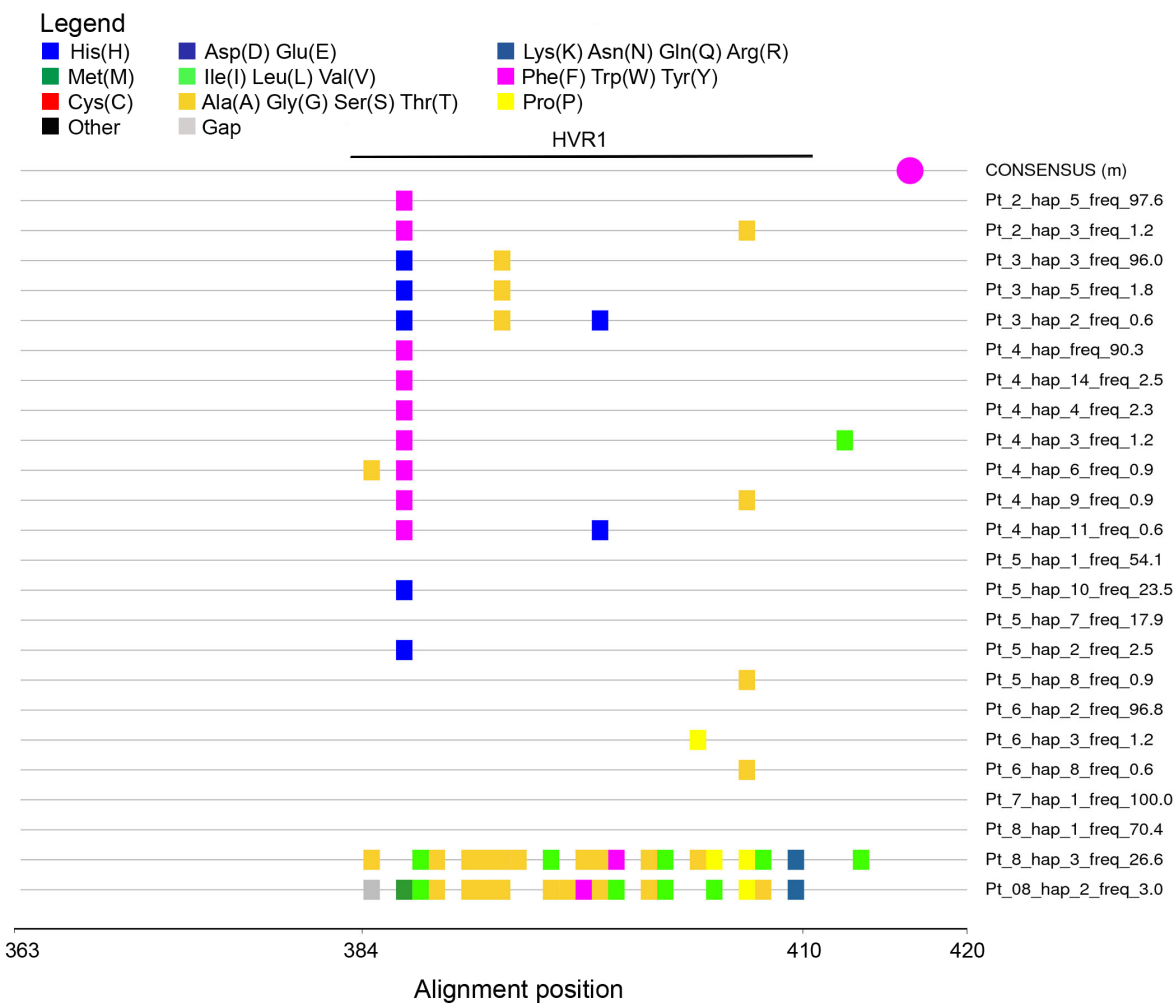
Highlighter plot showing differences in amino acid composition of HVR1 HCV sequence variants in hospitalized patients from the infection cluster. Variants are compared to consensus sequence built from all sequences present in all patients. Color coding of amino acid is shown in the legend. Potential N-glycosylation site in the consensus sequence is marked by a purple dot. Amino acid numbering follows the reference strain H77 (GenBank accession no. AF009606). HVR1 spans codons 384–410.

When analyzing amino acid substitutions compared to the consensus sequence, it was found that changes affected codons 384, 386, 392, 398, 404, 407 within the HVR1 and 413 outside the HVR1, whereas in variants 3 and 2 from patient 8 there were multiple changes (Fig 5). However, all the identified changes were outside of the potential N-glycosylation site at position 417.

## Discussion

In the present study we characterized HCV HVR1 variants in a group of patients from a regional hematology and oncology center. These patients were found to be HCV RNA positive after hospitalization but the source of infection remained unknown despite an extensive epidemiological investigation. As only one of the seven patients seroconverted at that time, the samples likely represented the very early stage of infection. We identified the presence of the same or nearly identical viral strain in all patients by phylogenetic linkage and by high sequence similarity of patients’ HVR1 variants (95.4% to 100.00%).

In five out of seven patients the phylogenetic analysis was either consistent with star-like phylogeny or there were no more than two variants present (one of which was dominant and the other was minor) which also suggests infection with a single founder. In addition, diversity per site within each patient viral population was low and roughly the same (except for patient 8), as would be expected by assuming that the divergence time since transmission was short and similar for each patient.

The complexity of variant populations, reflected by the number of nucleotide variants, was very low (mean 3.4 variants) which is in contrast to high diversity of HVR1 displayed during chronic infection. For example, in our previous study, the average number of HVR1 variants during chronic HCV 1b infection was 30–40 [16, 24]. This low complexity probably reflects the bottleneck effect at the time of transmission and suggests that the infection has been initiated by a single variant, so called “founder”, which must have been very similar or even identical to the consensus sequence inferred from all patients’ sequences [3, 4, 27]. Furthermore, the structure of HVR1 population was “narrow” in the majority of cases (as one predominant variant was accompanied by minor variants at <10% frequency), which is also compatible with a recent single variant infection. Previous studies showed that in chronic hepatitis C viral population tends to become more “flat” in terms of frequency structure, with higher predominance of moderate and low frequency variants [24]. Alternatively, the structure of the populations could have been affected by the presence of immunosuppression due to immunosuppressive drugs and the underlying disease. However, during such an early phase of infection the immune system response, which could narrow the population diversity is likely to be limited [4].

So far very few published studies analyzed diversity in the early stages of HCV infection [4–6, 28–32]. In the typical clinical setting, the complexity and diversity of HCV quasispecies is reduced at the time of transmission [6, 33]. However, a bottleneck may not be present in case of massive infections [30, 34].

In our study, the exact route of infection was not identified, but these could have been errors during line flushing and/or multidose vials use. In this case the inoculum (i.e. infectious dose) would have been very small which could explain the bottleneck and very “narrow” character of the viral lineages in the infected patients.

Whether one of the patients was the source of infection is unclear. Patient 8 differed from the other patients due to her higher intrahost HVR1 heterogeneity and high similarity of two of her strains to those found in the unrelated control. As these samples were sequenced in separate runs, using different multiplex sequence identifiers (MIDs) the findings were unlikely to be artifactual (sequencing or demultiplexing error or contamination). These data imply that patient 8 could have acquired the infection earlier and was subsequently superinfected with the predominant strain. Alternatively, the patient could have been the source of infection herself, transmitting only the predominant strain to other patients. Indeed, in the study of Campo et al, where multiple HCV infection outbreaks were studied by NGS, intrahost HVR1 populations derived from the infection source displayed the highest genetic heterogeneity [35].

Another possibility is that the source of infection was patient 5. This patient’s HVR1 variants displayed more complex phylogeny (higher number of clades) and frequency distribution which are typical for the later phase of infection (higher predominance of moderate and low frequency variants closely related to each other). Patient 5 was also the first to display elevation of ALT activity levels.

## Conclusions

Our NGS analysis of a cluster of HCV infections in the hospital setting revealed the presence of low diversity, very closely related variants in all patients, suggesting an early-stage infection with the same viral variant. NGS combined with phylogenetic analysis and classical epidemiological analysis could help in tracking of HCV outbreaks.

## References

[1] Farci P, Shimoda A, Coiana A, Diaz G, Peddis G, Melpolder JC, et al The outcome of acute hepatitis C predicted by the evolution of the viral quasispecies. Science. 2000;288(5464):339–44. . 10764648 10.1126/science.288.5464.339

[2] Domingo E, Sheldon J, Perales C. Viral quasispecies evolution. Microbiology and molecular biology reviews: MMBR. 2012;76(2):159–216. doi: 10.1128/MMBR.05023-11 ; PubMed Central PMCID: PMC3372249. 22688811 10.1128/MMBR.05023-11 PMC3372249

[3] Wang GP, Sherrill-Mix SA, Chang KM, Quince C, Bushman FD. Hepatitis C virus transmission bottlenecks analyzed by deep sequencing. J Virol. 2010;84(12):6218–28. doi: 10.1128/JVI.02271-09 ; PubMed Central PMCID: PMCPMC2876626. 20375170 10.1128/JVI.02271-09 PMC2876626

[4] Bull RA, Luciani F, McElroy K, Gaudieri S, Pham ST, Chopra A, et al Sequential bottlenecks drive viral evolution in early acute hepatitis C virus infection. PLoS pathogens. 2011;7(9):e1002243 doi: 10.1371/journal.ppat.1002243 ; PubMed Central PMCID: PMCPMC3164670. 21912520 10.1371/journal.ppat.1002243 PMC3164670

[5] Li H, Stoddard MB, Wang S, Blair LM, Giorgi EE, Parrish EH, et al Elucidation of hepatitis C virus transmission and early diversification by single genome sequencing. PLoS pathogens. 2012;8(8):e1002880 doi: 10.1371/journal.ppat.1002880 ; PubMed Central PMCID: PMCPMC3426529. 22927816 10.1371/journal.ppat.1002880 PMC3426529

[6] Brown RJ, Hudson N, Wilson G, Rehman SU, Jabbari S, Hu K, et al Hepatitis C virus envelope glycoprotein fitness defines virus population composition following transmission to a new host. J Virol. 2012;86(22):11956–66. doi: 10.1128/JVI.01079-12 ; PubMed Central PMCID: PMCPMC3486514. 22855498 10.1128/JVI.01079-12 PMC3486514

[7] Di Lello FA, Culasso AC, Campos RH. Inter and intrapatient evolution of hepatitis C virus. Ann Hepatol. 2015;14(4):442–9. . 26019029

[8] Thomas DL, Thio CL, Martin MP, Qi Y, Ge D, O'Huigin C, et al Genetic variation in IL28B and spontaneous clearance of hepatitis C virus. Nature. 2009;461(7265):798–801. doi: 10.1038/nature08463 ; PubMed Central PMCID: PMCPMC3172006. 19759533 10.1038/nature08463 PMC3172006

[9] Cochrane A, Searle B, Hardie A, Robertson R, Delahooke T, Cameron S, et al A genetic analysis of hepatitis C virus transmission between injection drug users. J Infect Dis. 2002;186(9):1212–21. Epub 2002/10/29. JID020314 [pii] doi: 10.1086/344314 . 12402190 10.1086/344314

[10] Gotz HM, van Doornum G, Niesters HG, den Hollander JG, Thio HB, de Zwart O. A cluster of acute hepatitis C virus infection among men who have sex with men—results from contact tracing and public health implications. AIDS. 2005;19(9):969–74. Epub 2005/05/21. 00002030-200506100-00015 [pii]. . 15905679 10.1097/01.aids.0000171412.61360.f8

[11] Liu Z, Netski DM, Mao Q, Laeyendecker O, Ticehurst JR, Wang XH, et al Accurate representation of the hepatitis C virus quasispecies in 5.2-kilobase amplicons. Journal of clinical microbiology. 2004;42(9):4223–9. doi: 10.1128/JCM.42.9.4223-4229.2004 ; PubMed Central PMCID: PMCPMC516368. 15365015 10.1128/JCM.42.9.4223-4229.2004 PMC516368

[12] Herring BL, Tsui R, Peddada L, Busch M, Delwart EL. Wide range of quasispecies diversity during primary hepatitis C virus infection. J Virol. 2005;79(7):4340–6. doi: 10.1128/JVI.79.7.4340-4346.2005 ; PubMed Central PMCID: PMCPMC1061543. 15767434 10.1128/JVI.79.7.4340-4346.2005 PMC1061543

[13] Keele BF, Giorgi EE, Salazar-Gonzalez JF, Decker JM, Pham KT, Salazar MG, et al Identification and characterization of transmitted and early founder virus envelopes in primary HIV-1 infection. Proc Natl Acad Sci U S A. 2008;105(21):7552–7. doi: 10.1073/pnas.0802203105 ; PubMed Central PMCID: PMCPMC2387184. 18490657 10.1073/pnas.0802203105 PMC2387184

[14] Prosperi MC, De Luca A, Di Giambenedetto S, Bracciale L, Fabbiani M, Cauda R, et al The threshold bootstrap clustering: a new approach to find families or transmission clusters within molecular quasispecies. PLoS One. 2010;5(10):e13619 Epub 2010/11/05. doi: 10.1371/journal.pone.0013619 ; PubMed Central PMCID: PMC2963616. 21049051 10.1371/journal.pone.0013619 PMC2963616

[15] Barzon L, Lavezzo E, Costanzi G, Franchin E, Toppo S, Palu G. Next-generation sequencing technologies in diagnostic virology. J Clin Virol. 2013;58(2):346–50. doi: 10.1016/j.jcv.2013.03.003 . 23523339 10.1016/j.jcv.2013.03.003

[16] Cortes KC, Zagordi O, Perlejewski K, Laskus T, Maroszek K, Bukowska-Osko I, et al Deep sequencing of hepatitis C virus hypervariable region 1 reveals no correlation between genetic heterogeneity and antiviral treatment outcome. BMC Infect Dis. 2014;14:389 doi: 10.1186/1471-2334-14-389 ; PubMed Central PMCID: PMC4226954. 25016390 10.1186/1471-2334-14-389 PMC4226954

[17] Helle F, Goffard A, Morel V, Duverlie G, McKeating J, Keck ZY, et al The neutralizing activity of anti-hepatitis C virus antibodies is modulated by specific glycans on the E2 envelope protein. J Virol. 2007;81(15):8101–11. doi: 10.1128/JVI.00127-07 ; PubMed Central PMCID: PMC1951279. 17522218 10.1128/JVI.00127-07 PMC1951279

[18] Helle F, Vieyres G, Elkrief L, Popescu CI, Wychowski C, Descamps V, et al Role of N-linked glycans in the functions of hepatitis C virus envelope proteins incorporated into infectious virions. J Virol. 2010;84(22):11905–15. doi: 10.1128/JVI.01548-10 ; PubMed Central PMCID: PMC2977866. 20844034 10.1128/JVI.01548-10 PMC2977866

[19] Burke KP, Cox AL. Hepatitis C virus evasion of adaptive immune responses: a model for viral persistence. Immunol Res. 2010;47(1–3):216–27. Epub 2010/01/13. doi: 10.1007/s12026-009-8152-3 ; PubMed Central PMCID: PMC2910517. 20066508 10.1007/s12026-009-8152-3 PMC2910517

[20] Caraballo Cortes K, Zagordi O, Laskus T, Ploski R, Bukowska-Osko I, Pawelczyk A, et al Ultradeep pyrosequencing of hepatitis C virus hypervariable region 1 in quasispecies analysis. Biomed Res Int. 2013;2013:626083 doi: 10.1155/2013/626083 ; PubMed Central PMCID: PMC3655449. 23710455 10.1155/2013/626083 PMC3655449

[21] Zagordi O, Bhattacharya A, Eriksson N, Beerenwinkel N. ShoRAH: estimating the genetic diversity of a mixed sample from next-generation sequencing data. BMC Bioinformatics. 2011;12:119 Epub 2011/04/28. doi: 10.1186/1471-2105-12-119 ; PubMed Central PMCID: PMC3113935. 21521499 10.1186/1471-2105-12-119 PMC3113935

[22] Tamura K, Peterson D, Peterson N, Stecher G, Nei M, Kumar S. MEGA5: molecular evolutionary genetics analysis using maximum likelihood, evolutionary distance, and maximum parsimony methods. Molecular biology and evolution. 2011;28(10):2731–9. doi: 10.1093/molbev/msr121 ; PubMed Central PMCID: PMC3203626. 21546353 10.1093/molbev/msr121 PMC3203626

[23] Tamura K, Nei M. Estimation of the number of nucleotide substitutions in the control region of mitochondrial DNA in humans and chimpanzees. Molecular biology and evolution. 1993;10(3):512–26. doi: 10.1093/oxfordjournals.molbev.a040023 . 8336541 10.1093/oxfordjournals.molbev.a040023

[24] Caraballo Cortes K, Zagordi O, Jablonska J, Pawelczyk A, Kubisa N, Perlejewski K, et al Spouse-to-Spouse Transmission and Evolution of Hypervariable Region 1 and 5' Untranslated Region of Hepatitis C Virus Analyzed by Next-Generation Sequencing. PLoS One. 2016;11(2):e0150311 doi: 10.1371/journal.pone.0150311 ; PubMed Central PMCID: PMC4769329. 26918636 10.1371/journal.pone.0150311 PMC4769329

[25] Hussein N, Zekri AR, Abouelhoda M, Alam El-Din HM, Ghamry AA, Amer MA, et al New insight into HCV E1/E2 region of genotype 4a. Virol J. 2014;11:231 doi: 10.1186/s12985-014-0231-y ; PubMed Central PMCID: PMCPMC4304183. 25547228 10.1186/s12985-014-0231-y PMC4304183

[26] McWilliam H, Li W, Uludag M, Squizzato S, Park YM, Buso N, et al Analysis Tool Web Services from the EMBL-EBI. Nucleic acids research. 2013;41(Web Server issue):W597–600. doi: 10.1093/nar/gkt376 ; PubMed Central PMCID: PMC3692137. 23671338 10.1093/nar/gkt376 PMC3692137

[27] Salazar-Gonzalez JF, Salazar MG, Keele BF, Learn GH, Giorgi EE, Li H, et al Genetic identity, biological phenotype, and evolutionary pathways of transmitted/founder viruses in acute and early HIV-1 infection. J Exp Med. 2009;206(6):1273–89. doi: 10.1084/jem.20090378 ; PubMed Central PMCID: PMC2715054. 19487424 10.1084/jem.20090378 PMC2715054

[28] Ray SC, Mao Q, Lanford RE, Bassett S, Laeyendecker O, Wang YM, et al Hypervariable region 1 sequence stability during hepatitis C virus replication in chimpanzees. J Virol. 2000;74(7):3058–66. ; PubMed Central PMCID: PMC111804. 10708420 10.1128/jvi.74.7.3058-3066.2000 PMC111804

[29] Ray SC, Fanning L, Wang XH, Netski DM, Kenny-Walsh E, Thomas DL. Divergent and convergent evolution after a common-source outbreak of hepatitis C virus. J Exp Med. 2005;201(11):1753–9. doi: 10.1084/jem.20050122 ; PubMed Central PMCID: PMC2213258. 15939791 10.1084/jem.20050122 PMC2213258

[30] Laskus T, Wilkinson J, Gallegos-Orozco JF, Radkowski M, Adair DM, Nowicki M, et al Analysis of hepatitis C virus quasispecies transmission and evolution in patients infected through blood transfusion. Gastroenterology. 2004;127(3):764–76. . 15362033 10.1053/j.gastro.2004.06.005

[31] Cantaloube JF, Biagini P, Attoui H, Gallian P, de Micco P, de Lamballerie X. Evolution of hepatitis C virus in blood donors and their respective recipients. J Gen Virol. 2003;84(Pt 2):441–6. doi: 10.1099/vir.0.18642-0 . 12560577 10.1099/vir.0.18642-0

[32] Escobar-Gutierrez A, Vazquez-Pichardo M, Cruz-Rivera M, Rivera-Osorio P, Carpio-Pedroza JC, Ruiz-Pacheco JA, et al Identification of hepatitis C virus transmission using a next-generation sequencing approach. Journal of clinical microbiology. 2012;50(4):1461–3. doi: 10.1128/JCM.00005-12 ; PubMed Central PMCID: PMC3318530. 22301026 10.1128/JCM.00005-12 PMC3318530

[33] D'Arienzo V, Moreau A, D'Alteroche L, Gissot V, Blanchard E, Gaudy-Graffin C, et al Sequence and functional analysis of the envelope glycoproteins of hepatitis C virus variants selectively transmitted to a new host. J Virol. 2013;87(24):13609–18. doi: 10.1128/JVI.02119-13 ; PubMed Central PMCID: PMC3838246. 24109215 10.1128/JVI.02119-13 PMC3838246

[34] Arenas JI, Gallegos-Orozco JF, Laskus T, Wilkinson J, Khatib A, Fasola C, et al Hepatitis C virus quasi-species dynamics predict progression of fibrosis after liver transplantation. J Infect Dis. 2004;189(11):2037–46. doi: 10.1086/386338 . 15143471 10.1086/386338

[35] Campo DS, Xia GL, Dimitrova Z, Lin Y, Forbi JC, Ganova-Raeva L, et al Accurate Genetic Detection of Hepatitis C Virus Transmissions in Outbreak Settings. J Infect Dis. 2016;213(6):957–65. doi: 10.1093/infdis/jiv542 ; PubMed Central PMCID: PMC5119477. 26582955 10.1093/infdis/jiv542 PMC5119477

